# Increased Serum Klotho With Age-Related Aortic Stiffness and Peripheral Vascular Resistance in Young and Middle-Aged Swine

**DOI:** 10.3389/fphys.2020.00591

**Published:** 2020-06-09

**Authors:** Xiaomei Guo, Ghassan S. Kassab

**Affiliations:** California Medical Innovations Institute, San Diego, CA, United States

**Keywords:** Klotho, aging, aortic stiffness, vascular resistance, femoral artery

## Abstract

The anti-aging function of Klotho gene has been implicated in age-related diseases. The physiological importance of Klotho in the progression of arterial stiffness with aging, however, remains unclear. The goal of this study is to determine the correlation of circulating Klotho with early age-related aortic stiffening and peripheral hemodynamics. We measured serum Klotho levels in a group of pigs with age ranges of 1.5–9 years and investigated the relationship between Klotho levels and biomarkers of aortic stiffening with aging, including aortic pulse wave velocity (PWV), augmentation index (AIx), and pulse pressure (PP). The effects of aortic stiffening on peripheral vascular resistance, compliance, and function were also evaluated. We found that increased aortic stiffness occurred at middle age (>5 years old), as evidenced by an increase in PWV and AIx (*p* < 0.001), but with no changes in blood pressure and PP. With advancing age, increased femoral vascular resistance positively correlated with aortic PWV and AIx (*p* < 0.01). No significant difference in endothelium function and arterial compliance for femoral and small peripheral arteries was observed between young and middle-aged groups. The serum Klotho levels were lower in young and higher in middle-aged pigs (*p* < 0.001), and a positive correlation was found between Klotho and aortic PWV, AIx, and femoral vascular resistance (*p* < 0.01). Our findings suggest that early-aged aortic stiffening has adverse effect on peripheral hemodynamics, independent of blood pressure levels. Elevated Klotho secretion was associated with increased aortic stiffness and peripheral vascular resistance with aging.

## Introduction

It is well known that aging is a potent, independent risk factor for cardiovascular disease (Greenwald, 2007; Mitchell et al., 2007). In response to aging, the central elastic arteries become progressively stiffer and their ability to absorb pulsations from the ejecting ventricle is reduced (Avolio et al., 1985; Kelly et al., 1989). Enormous epidemiological research has demonstrated that aortic stiffening increases pulsatile hemodynamic forces, which may exert a detrimental effect on peripheral vascular function, invoking a mechanism linking increased arterial stiffness to the age-related disorders (James et al., 1995; Mitchell et al., 2005). Although a marked age-related increase in aortic stiffness has been confirmed in normal subjects before 60 years of age (Mitchell et al., 2004, 2007), it is unclear whether peripheral vascular resistance and function change in response to early aortic aging. Hence, one goal of this study is to determine the relationship between early age-related aortic stiffening and peripheral hemodynamics in young and middle-aged swine.

Klotho gene was originally identified as an anti-aging protein, which encodes a single-pass transmembrane glycoprotein (Kuro-o et al., 1997; Shiraki-Iida et al., 1998). It is produced mainly in the kidney and the soluble form of Klotho is cleaved and released in the blood, urine, and cerebrospinal fluid (Imura et al., 2004; Moe and Drueke, 2008). In a transgenic mouse model, overexpression of Klotho by genetic manipulation of full-length Klotho resulted in a significant extension of life span (Kurosu et al., 2005). The deletion of Klotho gene in mice led to a premature aging syndrome (Kuro-o et al., 1997). In humans, circulating levels of soluble Klotho decrease with age (Xiao et al., 2004), and emerging evidence suggests that Klotho concentration is closely correlated to the development of cardiovascular disease (Semba et al., 2011; Hu et al., 2013) and chronic kidney disease (CKD) (Akimoto et al., 2012; Asai et al., 2012). Although the anti-aging function of Klotho in human aging and age-related disorders has been implicated (Xiao et al., 2004; Hu et al., 2013; Koyama et al., 2015), the biological role of circulating Klotho in the progression of arterial stiffness with the aging process has not yet been elucidated. The second goal of this study is to investigate the associations of serum Klotho with aortic stiffness and peripheral vascular resistance during aging from young to middle age in swine.

In this study, we chose swine as the experimental subject since swine is considered an ideal preclinical model due to the anatomical and physiological similarities in the cardiovascular system between swine and human (Swindle and Smith, 1998). We measured the serum Klotho concentrations in young and middle-aged swine and explored the possible connection between the serum Klotho level and signs of arterial stiffening with aging, including aortic pulse wave velocity (PWV), augmentation index (AIx), and pulse pressure (PP). The effects of early age-related aortic stiffening on peripheral vascular resistance, compliance, and function were also evaluated.

## Materials and Methods

### Experimental Animals

A total of 36 female Yucatan miniature pigs were divided into a young group (*n* = 18) with age ranges of 1.5–5 years and a middle-aged group (*n* = 18) with age ranges of 5.5–9 years (defined as middle age according to a life span of 15 years for Yucatan miniature swine). All animals were fed a standard swine diet (Teklad diet 7037, Envigo, Somerset, NJ) twice daily, and the amount of diet provided was 1% of the body weight of the pigs. All animal experiments were performed in accordance with national and local ethical guidelines, including the Institute of Laboratory Animal Research guidelines, Public Health Service policy, and the Animal Welfare Act, as approved by the Institutional Animal Care and Use Committee at California Medical Innovations Institute, San Diego.

### Blood Pressure Measurements and Pulse Wave Analysis

Pigs were fasted for 12 h prior to surgery and pre-anesthetized with TKX (Telazol 10 mg/kg, Ketamine 5 mg/kg, and Xylazine 5 mg/kg, i.m). Pigs were then anesthetized with 2–3% isoflurane inhalation. Ventilation with 100% O_2_ was provided with a respirator and maintained PCO_2_ at approximately 35 mmHg. Under fluoroscopic guidance (Philips Fluoroscopy System), an introducer sheath (6 Fr) was percutaneously inserted into the jugular vein for administration of 0.9% saline and heparin (100 IU/kg). A sheath (5 Fr) was used to introduce a catheter into the aortic arch (proximal site) *via* the carotid artery. Another sheath (5 Fr) was used to introduce a catheter into the abdominal aorta (distal site) *via* the right femoral artery. Those catheters were connected to a pressure transducer (PowerLab, ADInstruments Inc.). The thoracic (aorta arch) and abdominal aortic blood pressure waveforms were recorded from these two locations simultaneously during the procedure. The femoral blood pressure waveform was also recorded with a catheter placement at the left femoral artery *via* an introducer sheath (4 Fr).

Pulse wave analysis was applied to determine aortic stiffness. PWV calculation is based on the difference in arrival times of the pressure wave at the proximal (aorta arch) and distal (abdominal aorta) locations. Since the tips of pressure catheters placed on the proximal and distal sites are visible on radiographs, the propagation distance was obtained by measuring the distance between the two catheters. PWV (m/s) was calculated by dividing the propagation distance by the difference between the two arrival times (transit time). AIx is expressed as the difference between the second and first systolic peaks of central aortic waveform calculated as a percentage of PP.

### Femoral Flow Measurement and Analysis (Echocardiography)

The measurement of femoral blood flow velocity was obtained in all animals using an iE33 duplex ultrasonography (Philips, Andover, MA) equipped with L15-7 transducer. The mean velocity waveforms were recorded continuously for 6 s for further analysis. The diameter of the femoral artery was also recorded by B-mode imaging. According to the flow velocity waveforms, femoral flow volume (ml/min) was calculated from the time-averaged mean flow velocity and the arterial diameter. Femoral vascular resistance (mmHg/ml/min) was calculated by dividing the mean arterial pressure by the flow volume. After flow measurement, the femoral artery and a small peripheral artery (muscular branch of femoral artery with diameter 300–500 μm) were harvested for endothelial function and mechanical testing. Animal was then euthanized with a saturated solution of potassium chloride injection through the jugular vein to arrest the heart under deep anesthesia.

### Endothelial Function

An isovolumic myograph was used to evaluate the endothelium-dependent vasorelaxation (Lu et al., 2011). Briefly, the segments from femoral and small peripheral arteries were cannulated on both ends in a physiological bath with HEPES physiologic saline solution (HEPES-PSS, concentration in mmol/l: 142 NaCl, 4.7 KCl, 2.7 sodium HEPES, 3 HEPES acid, 0.15 NaHPO_4_, 1.17 MgSO_4_, 2.79 CaCl_2_, and 5.5 glucose, solution gassed by 95% O_2_ plus 5% CO_2_). Both vessels segments were stretched to *in situ* length and preloaded at the physiological pressure of 80 mmHg. The pressure and external diameter were measured with a pressure transducer (Mikro-Tip SPR-524; Millar Instruments) and a digital diameter tracking (DiamTrak v3+; Australia), respectively. The vessel segment was pre-constricted with phenylephrine (PE) by a series of doses (10^−10^–10^−5^ mol/L in the PSS), and then relaxed with acetylcholine (ACh) by a series of doses: 10^−10^–10^−5^ mol/L. The endothelium-independent relaxation to sodium nitroprusside (SNP, 10^−5^ mol/L) was measured to verify the sensitivity of vascular smooth muscle to nitric oxide (NO). The overall contractility of vessel was tested with potassium chloride (KCl) at 60 mmol/L. Contraction was expressed as percentage of the response to KCl. Relaxation was expressed as percentage of pre-contraction to PE.

### Mechanical Tests

The femoral and small peripheral arterial segments were cannulated on both ends and fully relaxed in Ca^2+^ free HEPES-PSS. The arterial segment was preconditioned with five cyclic changes in pressure from 0 to 180 mmHg. The pressure was then increased in 20 mmHg step increments from 20 to 180 mmHg in a staircase manner. The passive pressure (P)-diameter (D) relation was recorded. The lumen cross-sectional area (CSA) was computed from the lumen diameter (D), as CSA = πD^2^/4. The area compliance (C_CSA_) of the artery was determined by the slope of the pressure-CSA relationship, i.e., C_V_ = ∆CSA/∆P as described in a previous publication (Guo et al., 2014).

### Serum Klotho Level

Blood samples were collected in ethylenediaminetetraacetic acid (EDTA) tubes for all experimental pigs. After centrifugation at 5,000 rpm and 4°C for 15 min, serum was immediately separated and stored at −80°C until analysis. The circulating level of Klotho in the serum was measured by a Klotho ELISA kit (IBL Co. Ltd., Minneapolis, MN) according to the manufacturer’s guideline.

### Statistical Analysis

Results were shown as mean ± standard error of mean (mean ± SEM). Statistical analysis was performed using SigmaStat (Systat Software, California, USA). The significance of the differences between young and middle-age groups was evaluated by either *t*-test or one-way ANOVA. Multiple linear regression analysis was used to estimate the correlations between age and the study variables. Spearman’s correlation test was used to estimate the correlations between Klotho level and the study variables. Significant differences between the dose-dependent groups for endothelial function testing were determined by two-way ANOVA. The results were considered statistically significant when *p* < 0.05 (2-tailed).

## Results

**Figure 1:**
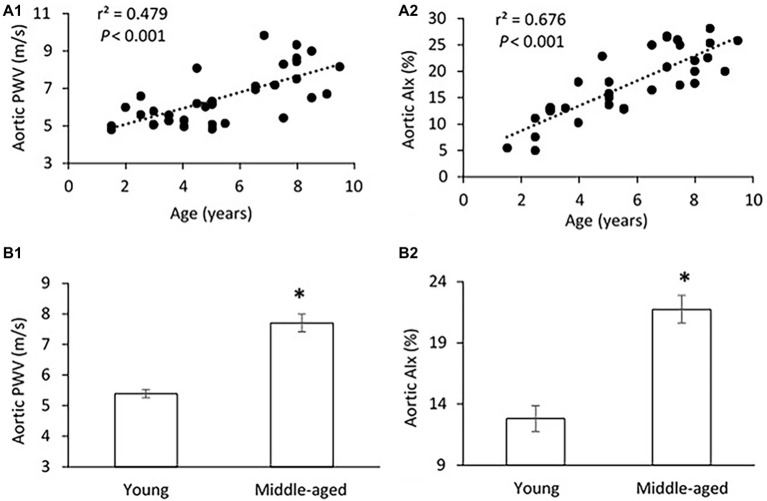
**(A)** Correlations of aortic pulse wave velocity (PWV) **(A1)** and augmentation index (AIx) **(A2)** with age. The linear regression analysis was conducted, and data are expressed as correlation coefficients and significance levels (*r*^2^; *p*). **(B)** Comparison of aortic PWV **(B1)** and AIx **(B2)** between young and middle-aged pigs. Data corresponds to means ± standard error of mean (mean ± SEM). *p* < 0.05, when compared to young group.

The mean age, body weight, hemodynamic parameters, and serum Klotho level were presented and compared between young and middle-aged groups in Table 1. The mean age is 3.5 ± 0.3 years (range of 1.5–5 years) for the young group and 7.2 ± 0.3 years (range of 5.5–9 years) for the middle-aged group. The two study groups have comparable values of body weight and heart rate. There was no between-groups difference in PP, systolic blood pressure (SBP), diastolic blood pressure (DBP), and mean arterial pressure (MAP) in thoracic and abdominal aortic pressure measures. Figure 1 shows a positive correlation of aortic PWV and AIx with age (*p* < 0.001) by using linear regression analysis. Aortic PWV and AIx was significantly increased in middle-aged group as compared to young group (*p* < 0.001). No correlation was found between blood pressure and PP with age (data not shown).

**Table 1:** Comparison of age, weight, hemodynamic parameters and serum Klotho levels between young and middle-aged pigs.

Variables	Young (*n* = 18)	Middle-aged (*n* = 18)	*p*
Age (years)	3.5 ± 0.3	7.2 ± 0.3	**<0.001**
Body weight (kg)	112.8 ± 3.3	116.3 ± 3.0	0.415
Heart rate (beats/min)	74.8 ± 2.5	71.3 ± 2.3	0.31
Thoracic aortic blood pressure (mmHg)			
Mean pressure	81.3 ± 2.7	77.0 ± 4.2	0.369
Systolic pressure	101.1 ± 2.6	97.6 ± 4.5	0.475
Diastolic pressure	71.3 ± 2.9	66.8 ± 4.1	0.333
Pulse pressure	29.7 ± 1.5	30.8 ± 1.4	0.592
Abdominal aortic blood pressure (mmHg)			
Mean pressure	78.7 ± 2.6	75.0 ± 4.1	0.411
Systolic pressure	105.8 ± 2.9	101.1 ± 4.9	0.372
Diastolic pressure	66.1 ± 2.7	62.9 ± 3.9.	0.36
Pulse pressure	39.7 ± 2.0	38.6 ± 2.4	0.805
Aortic PWV (m/s)	5.4 ± 0.1	7.7 ± 0.3	**<0.001**
Aortic AIx (%)	12.8 ± 1.2	21.7 ± 1.1	**<0.001**
Femoral blood pressure (mmHg)			
Mean pressure	70.8 ± 3.0.	72.8 ± 3.7	0.648
Systolic pressure	90.6 ± 4.3	92.7 ± 5.1	0.741
Diastolic pressure	60.8 ± 2.5	61.1 ± 3.6	0.952
Pulse pressure	29.8 ± 2.2	31.6 ± 2.4	0.559
Femoral artery diameter (mm)	4.02 ± 0.1	4.07 ± 0.5	0.727
Femoral mean flow rate (ml/min)	394.2 ± 21.1	277.1 ± 16.2	**<0.001**
Femoral vascular resistance (mmHg/ml/min)	0.19 ± 0.01	0.27 ± 0.017	**0.002**
Serum Klotho levels (pg/ml)	809.8 ± 193.6	2158.4 ± 164.5	**<0.001**

Values are mean ± SEM. Bold values are presented when p <0.05.

**Figure 2:**
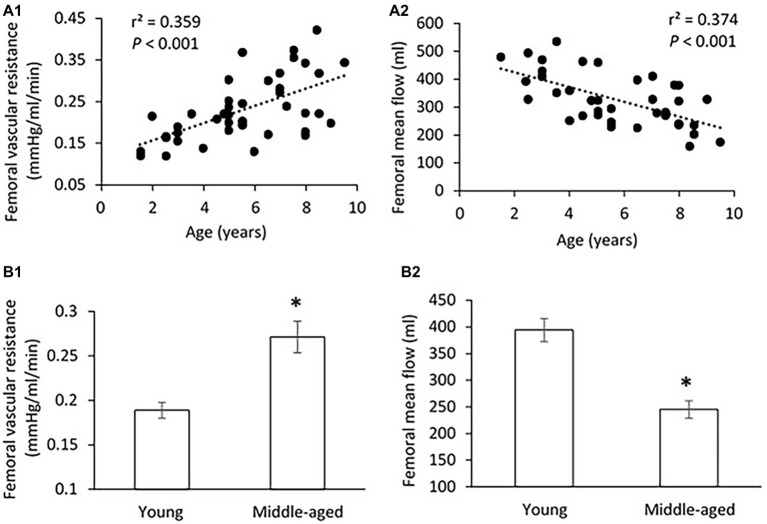
**(A)** Correlations of femoral vascular resistance **(A1)** and mean flow **(A2)** with age. The linear regression analysis was conducted, and data are expressed as correlation coefficients and significance levels (*r*^2^; *p*). **(B)** Comparison of femoral vascular resistance **(B1)** and mean flow **(B2)** between young and middle-aged pigs. Data corresponds to means ± SEM. *p* < 0.05, when compared to young group.

**Figure 3:**
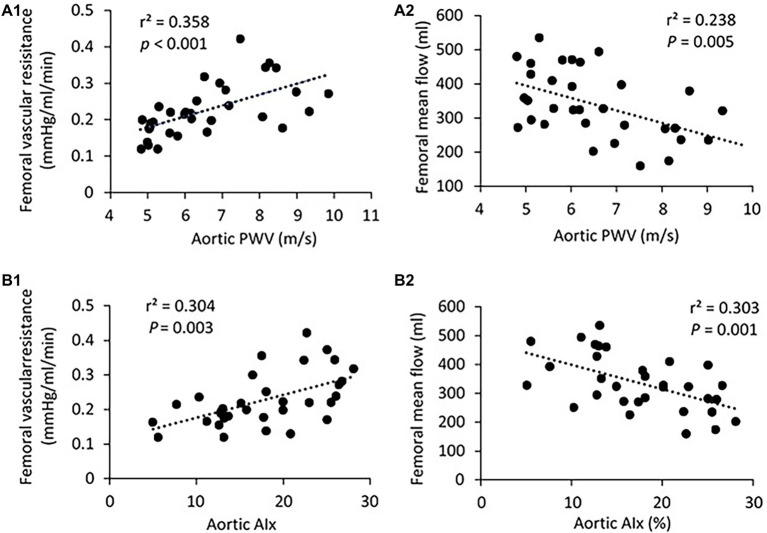
**(A)** Correlation of aortic PWV with femoral vascular resisitance **(A1)** and mean flow **(A2)**. **(B)** Correlation of aortic AIx with femoral vascular resisitance **(B1)** and mean flow **(B2)**. The linear regression analysis was conducted, and data are expressed as correlation coefficients and significance levels (*r*^2^; *p*).

There was no significant difference in PP, SBP, DBP, and MAP in the femoral pressure measures between young and middle-aged groups (Table 1). No difference in diameter of femoral artery was seen between these two groups. Figure 2 shows the correlation between vascular resistance and mean flow of femoral artery with age. The femoral vascular resistance was significantly increased (*p* < 0.01), whereas mean flow was significantly decreased (*p* < 0.01) in middle-aged as compared to young group. A linear relationship was observed between femoral vascular resistance and mean flow with age (*p* < 0.001). With regards to aortic stiffness, aortic PWV, and AIx were positively correlated with femoral vascular resistance (*p* < 0.01) and negatively correlated with femoral mean flow (*p* < 0.01) as shown in Figure 3.

**Figure 4:**
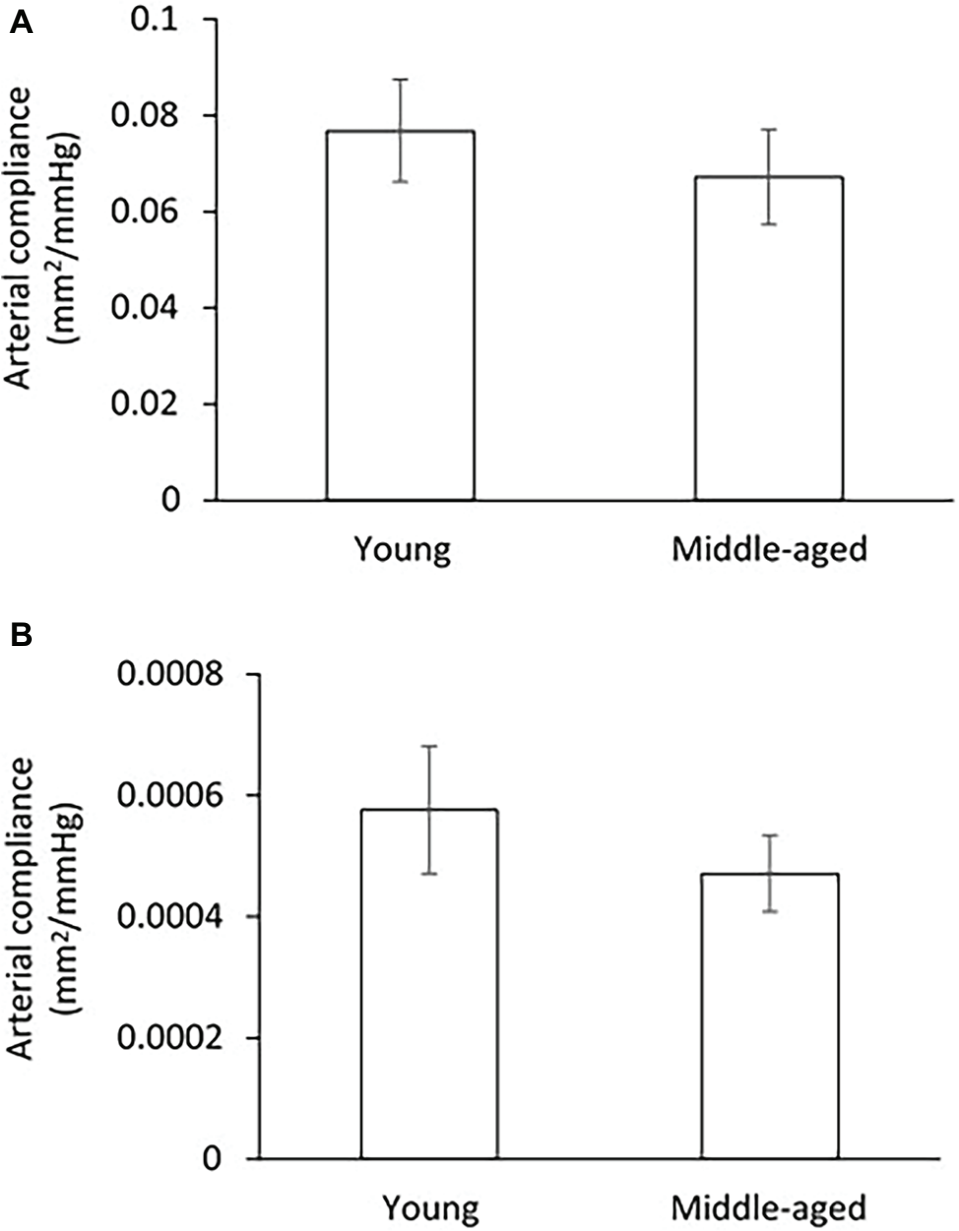
Comparison of arterial compliance for femoral artery **(A)** and small peripheral artery **(B)** between young and middle-aged pigs. Data corresponds to means ± SEM.

Compliance of arteries was presented as the pressure-cross-sectional area (P-CSA) relationship of the femoral artery and small peripheral artery. Although the middle-aged group has lower values of CSA compliance in both femoral and small peripheral arteries as compared to the young group (Figure 4), the differences are not statistically significant.

**Figure 5:**
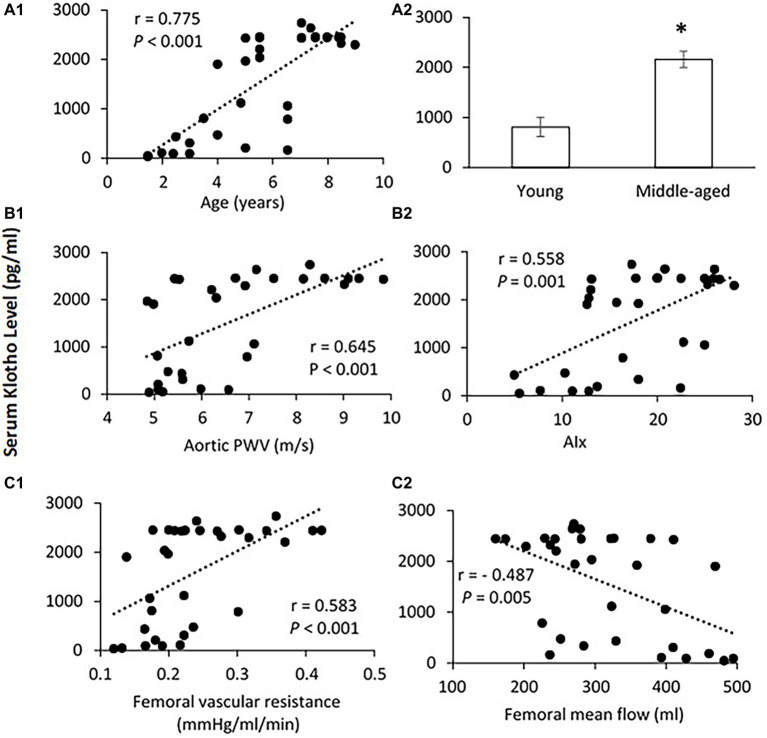
**(A)** Correlation of serum Klotho level with age **(A1)** and the mean levels of serum Klotho **(A2)** in young and middle-aged pigs. Data corresponds to means ± SEM. *p* < 0.05, when compared to young group. **(B)** Correlations of serum Klotho levels with aortic PWV **(B1)** and AIx **(B2)**. **(C)** Correlations of serum Klotho levels with femoral vascular resistance **(C1)** and mean flow **(C2)**. The Spearman’s correlation test was conducted, and data are expressed as correlation coefficients and significance levels (*r*; *p*).

Figure 5A shows the correlation between serum Klotho level and age. The mean Klotho level of the middle-aged group was significantly higher than that of the young-aged group (2,158.4 ± 164.5 vs. 809.8 ± 193.6 pg/ml, *p* < 0.001). The Spearman’s correlation test confirms that the serum Klotho level was positively correlated with age within the age ranges of 1.5–9 years (*p* < 0.001). There was a significantly positive correlation between serum Klotho levels and PWV (*p* < 0.001) and AIx (*p* = 0.001) as shown in Figure 5B. In Figure 5C, the Klotho level was also positively associated with femoral vascular resistance (*p* < 0.001), but slightly inversely correlated with femoral flow (*p* = 0.005). There was no apparent correlation of Klotho levels with aortic and femoral blood pressure and PP (data not shown).

**Figure 6:**
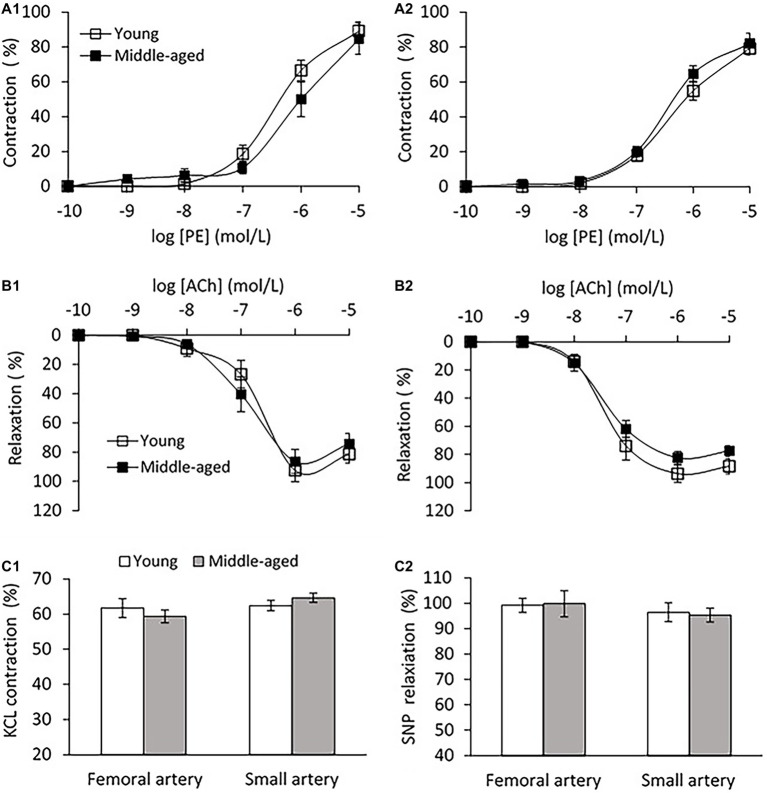
**(A)** Vascular contraction to phenylephrine (PE) in femoral artery **(A1)** and small peripheral artery **(A2)** between young and middle-aged pigs. **(B)** PE pre-contractile endothelium-dependent vasodilation to acetylcholine (ACh) in femoral artery **(B1)** and small peripheral artery **(B2)** between young and middle-aged pigs. **(C)** Vascular contraction to potassium chloride (KCL) at 60 mmol/L **(C1)** and maximal response of vasodilation to sodium nitroprusside (SNP) at 10^−5^ mol/L **(C2)** between young and middle-aged pigs in femoral and small peripheral artery. Data corresponds to means ± SEM.

Vascular endothelial function was evaluated by *ex-vivo* PE pre-contractile endothelium-dependent vasorelaxation. No significant difference in the vascular contractions to PE for both femoral and small peripheral arteries was observed between young and middle-age groups as shown in Figure 6A. For the femoral artery segment (4.04 ± 0.3 mm in diameter), the endothelium-dependent vasodilation in response to ACh was similar in middle-age groups compared with young group (Figure 6B1). For the small peripheral artery (410 ± 30 μm in diameter), the vasodilation to ACh in middle-age group tended to decrease, but the change was not significantly different from the young group (*p* = 0.08, Figure 6B2). In middle-aged group, the vascular contraction to KCl at 60 mmol/L did not show significant difference as compared to the young group for both femoral and small peripheral arteries (Figure 6C1). There was no significant difference in the maximal responses of endothelium-independent vasodilation to SNP at 10^−5^ mol/L between young and middle-aged groups for both arteries (Figure 6C2).

## Discussion

To our knowledge, this is the first study of the relation between Klotho and aortic stiffness and associated peripheral hemodynamics in swine. The major findings are as follows: (1) aortic PWV and AIx had a significant positive correlation with age in a group of pigs 1.5–9 years old; (2) mean aortic and femoral blood pressure, and PP did not differ between young (≤5 years) and middle age (>5 years) groups; (3) with advancing age, increased femoral vascular resistance, and decreased femoral flow were associated with aortic stiffening but with no changes in femoral endothelium function and arterial compliance; and (4) serum Klotho levels were lower in young and higher in middle-aged pigs, and positively correlated with aortic PWV, AI, and femoral vascular resistance.

Age-related arterial stiffening has emerged as an independent risk factor for cardiovascular events, including hypertension (Izzo, 2004), myocardial infarction (Mattace-Raso et al., 2006), diabetes (Cruickshank et al., 2002), heart failure (Chae et al., 1999), and stroke (Laurent et al., 2003). There is substantial evidence that aortic PWV and AIx predict cardiovascular morbidity and mortality in humans (Vlachopoulos et al., 2010). PWV represents the speed of a pressure wave propagating down a blood vessel, whereas AIx is a measure of the contribution made by the reflected pressure wave to the ascending aortic pressure waveform; both of which provide a measure of systemic arterial stiffness (Safar and London, 2000; Cecelja and Chowienczyk, 2012). Here, we found that aortic PWV and AIx significantly increased with age in 1.5–9 years old pigs. It has been extensively documented that arterial stiffness increases throughout the normal human life span (Mitchell et al., 2007) and is a major determinant of increased systolic and pulse pressure with advancing age (Kelly et al., 1989; Dernellis and Panaretou, 2005). In the middle-aged pigs, however, PP and SBP were not increased when compared with the younger pigs despite an increase in aortic stiffness. This result is consistent with some clinical observations that most of the age-related increase in PP occurs in elderly individuals (age > 60 years) and not in younger populations (Burt et al., 1995; Franklin et al., 1999). Although our data demonstrated that aging exerts a negative influence on aortic stiffness (raised PWV and AIx), even at middle age in healthy swine, whether age-related aortic stiffening precedes the development of elevated PP and/or SBP in elderly swine (>9 years old) is not known. This is a limitation of the current study because no swine older than 9 years were available from the vendors during the period of the experiment.

To answer the question of whether early aortic aging affects the hemodynamics of peripheral vasculature, we measured the vascular resistance, mean flow, and arterial compliance of femoral artery (average of 4.04 ± 0.3 mm in diameter range 3.2–4.8 mm) for all experimental pigs. We also measured the arterial compliance of small peripheral artery (average of 410 ± 30 μm in diameter range 308–520 μm). In a previous large community-based population study, without established hypertension or advanced vascular disease, a modest rise in forearm vascular resistance was reported to be related to increased aortic stiffness (Mitchell et al., 2005). Here, we found a marked increase in femoral vascular resistance with advancing age. It is reasonable to speculate that increasing peripheral vascular resistance in response to aortic stiffening would functionally reduce local flow, which was exhibited in middle-aged group as compared to young group by the use of ultrasonography measurements. To assess the relations between aortic stiffness and peripheral hemodynamics, we found that the femoral vascular resistance was positively correlated with the aortic PWV and AIx, indicating that early age-related aortic stiffening, even at normotensive PP or blood pressure levels, may have adverse influences on peripheral hemodynamics. In addition, arterial compliance, defined as the changes in luminal dimension (CSA) divided by the corresponding change in pressure, is conventionally thought to be a surrogate marker of arterial elasticity (Cohn et al., 2004). Our data showed no obvious changes in femoral and small peripheral arterial compliance between young and middle-aged groups, meaning a sustained peripheral arterial elasticity in ages 1.5–9 years of pigs. Similar results have been obtained in Framingham Heart Studies that the compliance or distensibility of large muscular arteries (i.e., femoral) is maintained at normal levels or changed relatively little despite an accelerated aortic stiffness with age (van der Heijden-Spek et al., 2000; Mitchell et al., 2004).

Dysfunction of the vascular endothelium has been associated with high blood pressure and arterial stiffening (Puddu et al., 2000; Safar et al., 2001). We examined the endothelium of femoral artery and found no evidence of endothelial dysfunction since comparable endothelium-dependent vasodilation to ACh was shown between young and middle-aged groups. For the small peripheral artery, although the vasodilation to ACh in middle-aged group has a descending trend, the change is not statistically significant compared with the young group (*p* = 0.08). There are emerging data that aortic stiffening may trigger remodeling, rarefaction, or hypertrophy in the microcirculation, leading to increased peripheral vascular resistance to blood flow (James et al., 1995; Liao et al., 2004). Several studies have provided direct evidence that functional and structural impairment of cerebral microcirculation are present in age-related cognitive decline (Toth et al., 2017) and Alzheimer’s disease (Csiszar et al., 2017). The current study only exhibited a downward trend in vascular function of peripheral muscular arteries with diameter >300 μm, we do not know whether abnormalities in microvasculature (<150 μm in diameter; Levy et al., 2001) occurs during the early-aged aortic stiffening, and whether microvascular dysfunction contributes to the increased peripheral vascular resistance in middle-aged swine. The mechanisms implicating the microcirculation need to be addressed in future studies.

Klotho is an aging suppressor gene whose protective properties are critical for a proper function of many tissues and organs (Kuro-o et al., 1997; Hu et al., 2013). A recent study by Semba et al. (2011) suggested that raised levels of Klotho were related to the lower prevalence of cardiovascular disease. More clinical research has reported that a deficiency of Klotho may be an early biomarker for CKD (Akimoto et al., 2012; Asai et al., 2012) and acute kidney injury (Hu et al., 2010). In this study, surprisingly, we found that the serum Klotho level was significantly increased in middle-aged group as compared to young group. Moreover, a positive relationship between Klotho level and age was seen in ages from 1.5 to 9 years of pigs. This result is contrary to the findings from human studies that circulating Klotho concentrations decreased remarkably with age (range of 0.1–88 years; Xiao et al., 2004; Yamazaki et al., 2010). Recently, Klotho protein was found to be expressed in human aorta (Lim et al., 2012) and coronary artery (Richter et al., 2016), which protects against endothelial dysfunction by increasing nitric oxide production (Saito et al., 1998, 2000). Moreover, the supplementation of exogenous Klotho attenuates oxidative stress, inflammation, and fibrosis by the inhibition of insulin/insulin-like growth factor-1 (IGF-1) and transforming growth factor-1 (TGF-1) signaling pathways to protect the vasculature and cardiac tissue (Yamamoto et al., 2005; Dalton et al., 2017; Takenaka et al., 2017). It is possible that an increase in serum Klotho level at middle age could be a compensatory response to the increased aortic stiffness with age to maintain endothelium function and arterial compliance of peripheral arterial system. Similar adaptive mechanism has been proposed by Paula et al. (2016) who observed an augmented level of Klotho in patients with myocardial infarction to ameliorate cardiac hypertrophy and remodeling. To the best of our knowledge, this study is the first to measure circulating Klotho concentrations with aging in swine, where species variations could be a potential factor that accounts for the different response of Klotho to advancing age between human and swine. The Spearman’s correlation analysis of our data showed that serum Klotho level was positively correlated with aortic PWV, AIx, and femoral vascular resistance, but not with blood pressure and PP. Our results confirmed a positive correlation of serum Klotho levels with age in young to middle-aged swine. Completion of further studies with the involvement of elderly swine (>9 years) is necessary to explore the pathophysiological mechanisms of serum Klotho fluctuation with advancing age. Whether Klotho gene provides vascular protection *via* upregulated Klotho production during early aortic aging remains to be determined as well.

There are limitations in the present study. Several clinical studies have reported that female is more susceptible to age-related cardiovascular events compared with male (Merz and Cheng, 2016; Rodgers et al., 2019). Here, we only focused on female swine with a range in ages 1.5–9 years. We did not evaluate sex variation in changes of serum Klotho and aortic stiffness with aging since no male swine >2 years old (and no female >9 years) were available from the vendors. It is recognized that different blood‐ and/or tissue-based biomarkers such as interleukin-6 (IL-6), tumor necrosis factor α (TNFα), and insulin-like growth factor 1 (IGF-1) are involved in vascular changes during aging (Tarantini et al., 2016; Justice et al., 2018). Most recently, a geroscience-guided clinical trial has suggested that the systematic evaluation of multi-biomarkers may be the best approach to clarify the biologic hallmarks of aging in human research (Justice et al., 2018). The current study, however, solely assessed a single blood-based biomarker “Klotho” which may not be able to reflect the complex and multifactorial processes underlying aging. In addition, we did not directly test the effect of serum Klotho on the aortic stiffness and peripheral vascular resistance. Hence, we cannot conclude any cause-and-effect relation. Our findings indicate an association between circulating Klotho and early age-related aortic stiffening and peripheral hemodynamics in swine.

In summary, early age-related aortic stiffening, even at normotensive PP or blood pressure levels, leads to functional alterations in peripheral hemodynamics, evidenced by increased femoral vascular resistance and reduced local flow. Elevated Klotho secretion with aging is associated with an increase in aortic stiffness and peripheral vascular resistance. Our findings support that aortic stiffening (increased PWV and AI) may be an early manifestation of aging. More research is required to investigate how Klotho interacts with the mechanisms of arterial stiffness in old swine. These studies serve as fundamental reference for the swine translational model in understanding human disease and testing of potential therapeutics.

## Data Availability Statement

The raw data supporting the conclusions of this article are available on request to the corresponding author.

## Ethics Statement

The animal study was reviewed and approved by Institutional Animal Care and Use Committee at California Medical Innovations Institute, San Diego.

## Author Contributions

XG conducted the experiments. XG collected and analyzed the data. XG and GK designed the experiments and revised the manuscript.

## Conflict of Interest

GK is the founder of 3DT Holdings. GK had the involvementwith the study design and preparation of the manuscript.The remaining author declares that the research was conducted in the absenceof any commercial or financial relationships that could be construed as a potentialconflict of interest.
